# Structural and functional asymmetry of RING trimerization controls priming and extension events in TRIM5α autoubiquitylation

**DOI:** 10.1038/s41467-022-34920-3

**Published:** 2022-11-19

**Authors:** Frank Herkules, Corey H. Yu, Alexander B. Taylor, Vi Dougherty, Susan T. Weintraub, Dmitri N. Ivanov

**Affiliations:** Department of Biochemistry and Structural Biology, UT Health San Antonio, San Antonio, TX 78229 USA

**Keywords:** Structural biology, Enzyme mechanisms, Innate immunity

## Abstract

TRIM5α is an E3 ubiquitin ligase of the TRIM family that binds to the capsids of primate immunodeficiency viruses and blocks viral replication after cell entry. Here we investigate how synthesis of K63-linked polyubiquitin is upregulated by transient proximity of three RING domains in honeycomb-like assemblies formed by TRIM5α on the surface of the retroviral capsid. Proximity of three RINGs creates an asymmetric arrangement, in which two RINGs form a catalytic dimer that activates E2-ubiquitin conjugates and the disordered N-terminus of the third RING acts as the substrate for N-terminal autoubiquitylation. RING dimerization is required for activation of the E2s that contribute to the antiviral function of TRIM5α, UBE2W and heterodimeric UBE2N/V2, whereas the proximity of the third RING enhances the rate of each of the two distinct steps in the autoubiquitylation process: the initial N-terminal monoubiquitylation (priming) of TRIM5α by UBE2W and the subsequent extension of the K63-linked polyubiquitin chain by UBE2N/V2. The mechanism we describe explains how recognition of infection-associated epitope patterns by TRIM proteins initiates polyubiquitin-mediated downstream events in innate immunity.

## Abstract (web summary)

TRIM5α is an E3 ligase that inhibits retroviral replication. Here, the authors delineate the biochemical mechanism that accelerates N terminal autoubiquitylation of TRIM5α upon its association with the retroviral capsid and, thus, enables recognition of an infection-associated molecular pattern.

## Introduction

Protein ubiquitylation is an essential mechanism of the proteome’s posttranslational regulation that contributes to virtually every facet of eukaryotic biology^1^. The versatility and richness of ubiquitin-mediated regulation are primarily controlled at the level of E3 ubiquitin ligases that catalyze ubiquitin transfer from high-energy thioester-linked E2~Ub conjugates (~ denotes thioester bond) onto substrate proteins^2^. Understanding how E3 ubiquitin ligases select their substrates and how E3 activity is regulated is a rapidly evolving area of biomedical research.

**Fig. 1:**
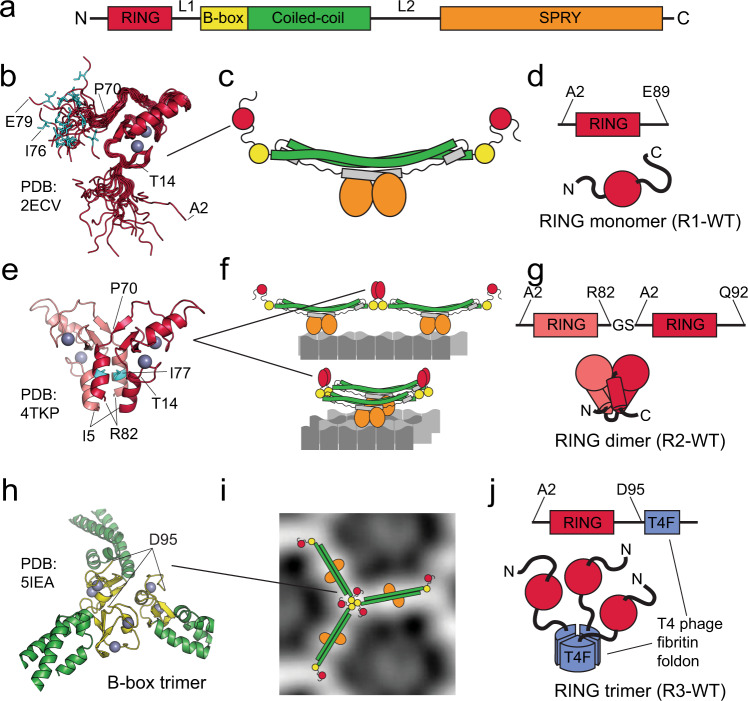
Recombinant RING constructs mimic distinct relative RING arrangements in TRIM5α. **a** Conserved tripartite motif (TRIM) domain arrangement in TRIM5α. **b** NMR structure of the human TRIM5α RING domain monomer (PDB: 2ECV). **c** Cartoon representation of the TRIM5α dimer, in which the two RING domains are presumed to exist as monomers. **d** The monomeric R1-WT RING construct used in this study. **e** Crystal structure of the rhesus TRIM5α RING (PDB: 4TKP) reveals the four-helix-bundle dimer interface. The isoleucine residue in the core of the four-helix bundle is highlighted in cyan here (I77) and in panel **a** (I76). **f** RING dimerization is thought to occur when multiple TRIM dimers come into proximity upon association with their binding partners. **g** RING dimerization is stabilized in the R2-WT tandem RING construct. **h** Crystal structure of the B-box trimer (PDB: 5IEA) reveals the structural arrangement of three B-box domains at the vertices of the honeycomb-like TRIM5α assemblies (**i**)^19, 26^. **j** Proximity of three RING domains at the vertices of the TRIM5α honeycomb was mimicked in the R3-WT construct by fusing RING domain to the T4 fibritin foldon, a compact trimerization domain.

Tripartite motif (TRIM) proteins constitute a large sub-family of RING E3 ligases defined by the shared domain arrangement, in which an N-terminal RING domain is followed by B-box and coiled-coil domains^3–6^ (Fig. 1a). This conserved domain pattern and the resulting structural similarities among TRIM proteins are thought to reflect similarities in the molecular mechanisms controlling their E3 activity, whereas functional versatility of TRIM proteins arises from differences in the composition of their C-terminal regions that mediate interactions with distinct binding partners^6,7^. The emerging picture is that the E3 activity of TRIM proteins is controlled by the interaction of their C-terminal domains with the cognate cellular ligands in a way that goes beyond simple selection and recruitment of ubiquitylation targets. In this study, we investigate how higher-order assembly of TRIM5α on the surface of retroviral capsids promotes activation and autoubiquitylation of this TRIM ubiquitin ligase.

TRIM5α is one of the most extensively studied members of the TRIM family, owing to its role in determining host tropism of primate immunodeficiency viruses, most notably—HIV^8–10^. The C-terminal SPRY domain of TRIM5α has evolved to bind retroviral capsids and its high variability among primates is thought to reflect the ongoing evolutionary antagonism between primate immunodeficiency viruses and their hosts^11–18^. The mechanism of the TRIM5α-mediated block to retroviral infectivity is not fully understood, but it is well established that it depends on direct binding of TRIM5α to mature retroviral cores upon their entry into the cytoplasm of the target cell^11,12^. A seminal electron-microscopy study revealed that interaction of TRIM5α with the regular pattern of binding epitopes present on the surface of the retroviral capsid promotes higher-order assembly of TRIM5α into honeycomb-like structures templated by the fullerene-like architecture of the capsid^19^. Structural studies on the individual domains of TRIM5α offered atomic resolution insight into this process^10^. Free TRIM5α in solution forms rod-shaped dimers held together by the long anti-parallel coiled-coil segment, the structural feature likely shared by most members of the TRIM family^20,21^. The coiled-coil rods are thought to function as molecular rulers of sorts that determine the preference of TRIM5α for particular spacing between distinct binding epitopes^22^. The N-terminal domains, RING and B-box, of each monomer reside almost 20 nm apart in the dimer on the opposite ends of the anti-parallel coiled-coil. In the capsid-templated higher-order assemblies of TRIM5α, individual dimer rods associate at the vertices of the honeycomb lattice through interactions mediated by the B-box domains^19,23–27^. There is general consensus based on an extensive body of evidence that the visually arresting higher-order structures formed by TRIM5α, and the pattern recognition functionality that they imply, are indispensable for the antiviral activity of the protein^10^.

In contrast to the critical role played by the higher-order assembly, the contribution of the E3 activity to the retroviral restriction by TRIM5α is less well understood. The E3 activity is required for accelerated viral uncoating and the inhibition of reverse transcription by TRIM5α, but, surprisingly, RING mutations and proteasome inhibitors do not abrogate the infectivity block, even though they restore normal uncoating and reverse transcription^28–34^. These puzzling findings suggest that TRIM5α may create multiple independent blocks to viral infectivity. The only known ubiquitylation substrate of TRIM5α is the protein itself and the autoubiquitylation results in the relatively short lifetime of the protein in the cell^30,34^. The rapid turnover is further enhanced by retroviral infection, which suggests that binding to the retroviral capsids upregulates autoubiquitylation^35^. One potential explanation of how the E3 activity contributes to the antiviral function emerged from the discovery that synthesis of K63-linked polyubiquitin by TRIM5α activates TAK1 and contributes to NF-κB-mediated signaling^36^. The findings suggested that TRIM5α may act as an innate immune sensor for the retrovirus capsid lattice. This and subsequent work revealed that TRIM5α-mediated RT inhibition and innate signaling depend on two specialized E2 proteins, UBE2W and UBE2N, and implicated the N-terminal autoubiquitylation of TRIM5α with K63-linked polyubiquitin chains in its antiviral activity^36–38^. Antibody-dependent intracellular neutralization of viruses by another TRIM family member, TRIM21, was shown to similarly require UBE2W- and UBE2N-mediated autoubiquitylation^39–41^. Dependence of TRIM5α and TRIM21 on two distinct E2s for priming and extension of the polyubiquitin chain bears resemblance to E3 mechanisms previously described for other, non-TRIM, ubiquitin ligases^42–45^. Collectively, these observations motivated the efforts to better understand the mechanism that couples capsid-templated assembly of TRIM5α to its E3 ubiquitin ligase activity and autoubiquitylation.

The first insight into how TRIM5α autoubiquitylation could be promoted by the association with the capsid emerged from the finding that polyubiquitin synthesis by TRIM5α depends on the dimerization of the RING domains^46^. Independently, RING dimerization was also shown to mediate the E3 activity of TRIM25^47,48^. RING dimerization, which activates E2~Ub conjugates by stabilizing a particular relative arrangement of the E2 and Ub^49–51^, is difficult to envision within isolated TRIM dimers because RING domains are located on the opposite ends of the long anti-parallel coiled-coil segment. Instead, RING dimerization is thought to occur when multiple TRIM dimers come together by binding to distinct binding epitopes located in proximity to each other and associate into higher-order structures through B-box interactions^46–48^. However, in the case of TRIM5α, not two, but three RING domains from three distinct TRIM5α dimers are brought into proximity by the interactions of the B-box domains at the vertices of the honeycomb-like lattice^19,23,26,27^, which raises questions about the functional relationship between B-box trimerization and RING dimerization. Initial evidence that RING dimerization is not sufficient for NF-κB activation emerged from the study by Fletcher et al., which suggested that three RING domains are necessary for K63-linked extension of TRIM5α-conjugated polyubiquitin^38^. Other recent studies established that the autoubiquitylation of TRIM21 is mediated by a very similar mechanism^40,52–54^.

In this study we use NMR spectroscopy, FRET ubiquitin discharge assays, and other approaches to delineate the biochemical basis of TRIM5α E3 activation by capsid binding. Using series of engineered TRIM5α constructs that mimic different RING oligomerization states we show why proximity of at least three RING domains is required to accelerate UBE2W-mediated priming and UBE2N-mediated extension in the autoubiquitylation of TRIM5α.

## Results

### Recombinant constructs were designed to mimic distinct RING oligomerization states of TRIM5α

We set out by generating series of recombinant RING constructs that mimic different relative arrangements of RING domains—monomer, dimer and trimer—thought to occur in the distinct oligomerization states of TRIM5α.

The isolated TRIM5α RING domain is predominantly monomeric at low concentrations^46^, and NMR studies revealed that the N-terminal and C-terminal segments of the RING domain, which flank the Zn-coordinating core, are largely unstructured in solution (PDB: 2ECV; Fig. 1b)^34^. In contrast, once crystallized, TRIM5α RING domains were found to form dimers, in which the N-terminal and C-terminal segments flanking the Zn-binding core associate into a four-helix bundle with hydrophobic interior (Fig. 1e)^46^. This dimerization mode is highly conserved in the TRIM family as it has now been observed in all known crystal structures of TRIM RINGs (TRIM5α: 4TKP^46^, TRIM37: 3LRQ, TRIM69: 6YXE^55^, TRIM23: 5VZW^56^, TRIM32: 5FEY^47^, TRIM25: 5EYA^47,48^, TRIM21: 6S53^54^ and possibly others).

To gain further insight into transient RING dimerization we took advantage of the two constructs used in our previous work, an isolated RING monomer (R1-WT) and a tandem RING dimer (R2-WT)^46^. The R1-WT construct encompasses residues 2–89 of the rhesus TRIM5α. This construct contains all residues needed for RING dimerization but remains predominantly monomeric at low concentrations in solution. The monomeric state of this construct is likely to represent the predominant conformational state of the RING domain within isolated TRIM5α dimers, because the two RINGs are held almost 20 nm apart on the opposing ends of the anti-parallel coiled-coil and cannot dimerize within the TRIM5α dimer (Fig. 1c). Weak dimerization of isolated RING domains is often stabilized by creating a tandem RING fusion construct^50^. We used this strategy to prepare a rhesus TRIM5α tandem construct, R2-WT, in which residue R82 of the first RING is linked to residue A2 of the second through a dipeptide Gly-Ser linker (Fig. 1g).

Finally, to mimic proximity of three RING domains promoted by B-box trimerization at the vertices of the honeycomb-like TRIM5α assemblies we prepared a trimeric RING construct (R3-WT), in which the RING domain (residues 1–95) is fused at the C-terminus to the T4 fibritin foldon, a 27-residue peptide that forms tight trimers and is often used to create or stabilize trimeric proteins (Fig. 1h–j)^57^. In the T4 foldon trimer the N-termini of each monomer are located on one face of the trimer approximately 12 Å from each other, which makes it a reasonably good mimic of the B-box trimer, whose N-termini (residue D95) are located 22 Å angstroms apart in the crystal structure^26^. The unstructured linker between RING and B-box domains (residues 83–95) is sufficiently long to make differences in the spacing between N-termini of the T4 foldon and B-box trimer insignificant.

RING dimerization can be disrupted by introducing a point mutation that replaces an isoleucine residue in the interior of the four-helix bundle of the dimer (I77 in the rhesus, I76 in the human TRIM5α) with an arginine (I77R)^46^. This mutation is expected to disrupt the four-helix bundle and RING dimerization, but to have only a minor effect on the monomeric form of the RING domain, in which I77 should be exposed to the solvent (Fig. 1b, e). In this study we introduced the I77R point mutation into the R1-WT and R3-WT constructs, yielding R1-I77R and R3-I77R constructs, respectively. Finally, we also prepared a trimeric RING construct (Ub-R3-WT), in which the full-length WT ubiquitin sequence is cloned immediately preceding the residue A2 of the RING in R3-WT, yielding the fusion protein that is chemically identical to the product of N-terminal monoubiquitylation of R3-WT by the UBE2W. In summary, six recombinant RING domain constructs (R1-WT, R1-I77R, R2-WT, R3-WT, R3-I77R, and Ub-R3-WT) were investigated here to establish the structural and biochemical determinants of TRIM5α E3 activity.

### Proximity of three RINGs creates an asymmetric arrangement, in which two RINGs form a compact dimer and the third remains in a monomer-like state with an unstructured N-terminal segment

Recombinant RING constructs were first investigated by sedimentation velocity analytical ultracentrifugation to ascertain that they form the desired oligomeric states (Supplementary Fig. 1A). The R2-WT and R3-WT constructs sedimented at the rates that were in good agreement with the theoretically predicted values based on their molecular weights and expected oligomerization states. Sedimentation velocity analysis of the R1-WT sample revealed that the sample exists as a mixture of the monomer and the dimer. In contrast, no detectable dimer component was present in the R1-I77R sample, as expected. To gain further insight into the monomer-dimer equilibrium of the R1-WT samples, we carried out series of sedimentation equilibrium experiments performed at different protein concentrations and rotor speeds. Fitting the entire dataset using the simple monomer-dimer equilibrium model yielded the dimerization constant K_D_ = 63 ± 28 μM with good residuals except in the lowest concentration samples (Supplementary Fig. 1B).

**Fig. 2:**
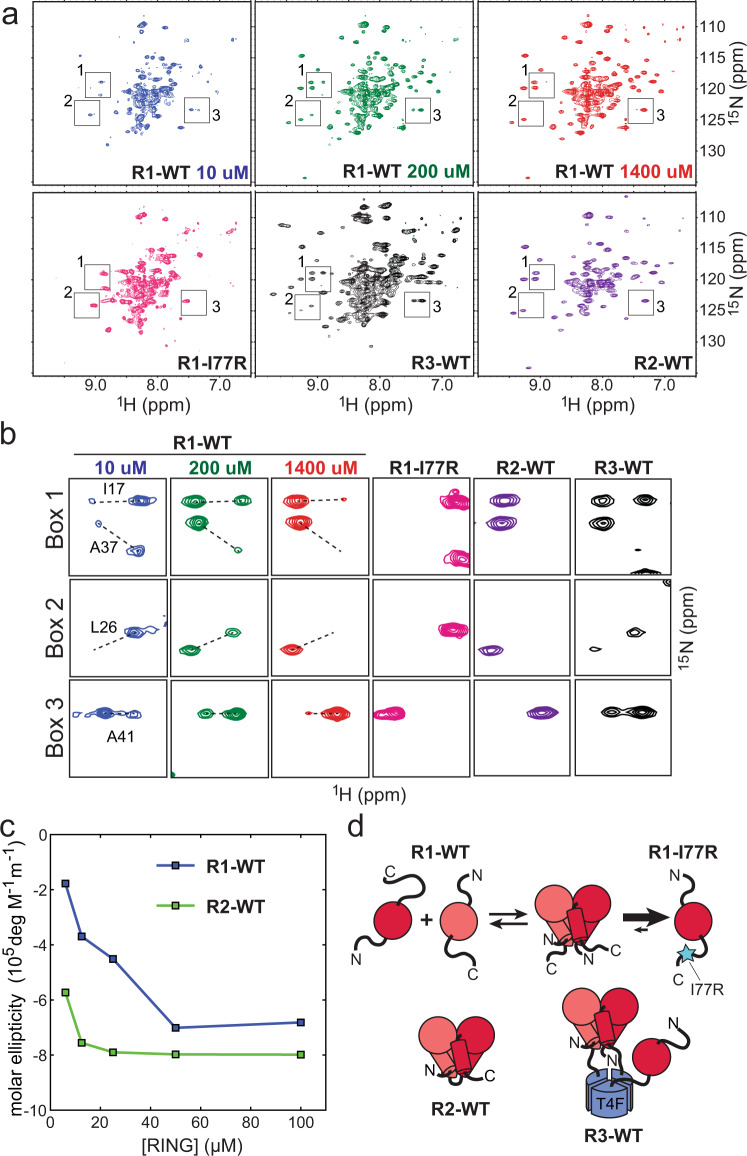
Conformational repertoire of distinct RING constructs as revealed by NMR spectroscopy and circular dichroism. **a** ^15^N-TROSY HSQC spectra of different RING constructs reveal spectral signatures of RING dimerization. **b** Three representative spectral regions from (**a**) showing NMR signals that display distinctive chemical shift changes upon transition between the monomeric and the dimeric states of the RING domain. **c** Dependence of molar ellipticity on protein concentration reveals that almost three-quarters of total alpha-helical content is lost in the R1-WT construct as protein concentration is lowered from 100 μM to 6 μM (*n* = 2 independent experiments). **d** Cartoon representation of the distinct conformational and oligomeric states observed in different RING constructs.

We then investigated RING conformational states in different constructs by NMR spectroscopy. ^15^N HSQC spectra of R1-WT, R2-WT, and R1-I77R samples collected at high protein concentration revealed that the R1-WT and R2-WT spectra were remarkably similar to each other with most peaks displaying a near perfect overlap (Fig. 2a). In contrast, the R1-I77R spectrum was dramatically different with significantly lower overall dispersion and higher signal crowding in the central area of the spectrum, which is indicative of significantly higher disorder present in the R1-I77R sample compared to R1-WT and R2-WT. We then collected series of ^15^N HSQC spectra by gradually decreasing protein concentration of the R1-WT sample from 1400 μM to 10 μM. As the concentration of the sample was lowered one set of peaks became progressively weaker whereas a new set of peaks appeared and became dominant (Fig. 2a, b). At 10 μM the R1-WT spectrum became strikingly similar to that of the R1-I77R sample. The findings confirmed that the R1-WT construct exists in a dimer-monomer equilibrium and that the two distinct sets of NMR signals present at high and low concentrations correspond to the dimeric and monomeric states of the protein, respectively. In addition, the similarity of the low-concentration R1-WT and R1-I77R spectra revealed that the I77R mutation does not significantly perturb the structure of the monomeric state of R1-WT. The dimer-monomer exchange rate is slow on the NMR time scale (<10 Hz) which indicates that the dimer association rate is slow in agreement with a major conformational change taking place upon dimerization. Notably, the spectra of the R3-WT sample contained both sets of NMR signals, corresponding to the monomer and the dimer, which revealed that when three RING domains are brought together, two RINGs form the dimer observed in the crystal structure, whereas the third RING remains in a monomer-like state (Fig. 2d).

Finally, we used circular dichroism (CD) spectroscopy to quantify the alpha-helical content in the R1-WT and R2-WT constructs. Series of CD spectra were collected and molar ellipticities at 222 nm were calculated and plotted as a function of protein concentration (Fig. 2c). Molar ellipticity of the R1-WT construct was strongly dependent on protein concentration, and approximately 75% of alpha-helical content was lost as protein concentration was lowered from 100 μM to 6 μM. In contrast, the ellipticity of the R2-WT construct was reduced only 30% in the lowest concentration sample. The alpha-helical content of the R1-WT construct was approximately 85% that of the R2-WT at 100 μM, but only about 30% at 6 μM. The CD results provide an independent confirmation of the structural changes occurring in the RING domain concomitantly with its dimerization and demonstrate that a large fraction of alpha-helical content is lost upon dissociation of the RING dimer into monomers. The observations confirm that order-disorder transitions are taking place in the N-terminal and C-terminal segments of the RING domain, which form a four-helix bundle in the dimer but appear largely disordered in the monomeric state (Fig. 2d). This conclusion is also supported by the NMR data in Fig. 2a, b that reveal high structural similarity between the monomeric state of R1-WT and the R1-I77R mutant, in which the four-helix bundle is disrupted.

### A FRET-based ubiquitin discharge assay reveals that RING dimerization is required for activation of UBE2N~Ub/V2 and UBE2W~Ub conjugates for ubiquitin transfer

**Fig. 3:**
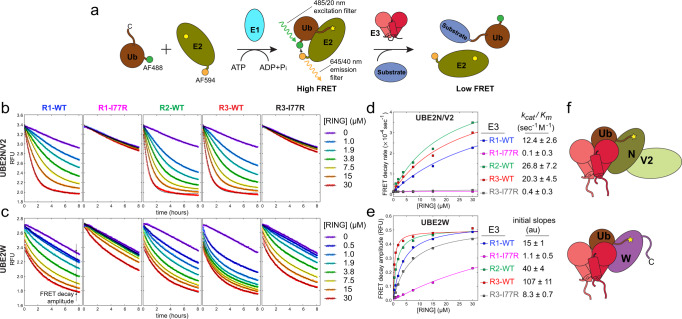
The contribution of RING dimerization to the catalytic enhancement of ubiquitin discharge from the UBE2N~Ub and UBE2W~Ub conjugates can be quantified using a FRET ubiquitin discharge assay. **a** A cartoon schematic of the FRET ubiquitin discharge assay used in this study. **b** Kinetics of RING-catalyzed ubiquitin discharge from the heterodimeric UBE2N~Ub/V2 (0.1 µM) is well approximated by an exponential decay function in agreement with the Michaelis-Menten equation (Supplementary Methods). **c** In contrast, ubiquitin discharge from UBE2W~Ub (0.1 µM) is not well described by the Michaelis-Menten model. **d** UBE2N/V2 ubiquitin discharge rates are plotted against RING concentration and the initial slopes (k_cat_/K_m_) can be used for quantification of the catalytic activity (*n* = 2 independent experiments). **e** Quantification of UBE2W discharge (see “Methods” and Supplementary Methods for more details) establishes that UBE2W is also activated by RING dimerization, albeit less potently than UBE2N. [RING] concentration is shown here as total concentration of RING domains in each sample irrespective of the oligomerization state (e.g., 15 μM sample of the tandem RING construct R2-WT has [RING] = 30 μM) (*n* = 2 independent experiments). **f** Cartoon representation of the dimerization-dependent activation of both UBE2N~Ub and UBE2W~Ub by TRIM5α RING.

To evaluate contributions of distinct RING oligomeric states to TRIM5α autoubiquitylation we investigated the catalytic activity of the RING constructs. E3 ubiquitin ligases catalyze the transfer of ubiquitin from the high-energy thioester-linked E2~Ub conjugates onto an amine group of the substrate. One of the challenges in the mechanistic studies of E3 ligases is the difficulty of measuring rates of chemical transformations catalyzed by E3 ligases with sufficient precision. In this study we developed a FRET-based ubiquitin discharge assay that allows continuous, real-time measurement of E3-catalyzed ubiquitin release from E2~Ub conjugates present at nanomolar concentrations in multiple samples in parallel using a fluorescence plate reader (Fig. 3a). To this end, different E2s and ubiquitin were N-terminally labeled with acceptor (AF594) and donor (AF488) fluorescent dyes, respectively, using sortase-catalyzed transpeptidation (Supplementary Fig. 2A, B)^58^. AF594-E2s were conjugated to AF488-ubiquitin using recombinant E1 enzyme in a standard procedure and the conjugation reactions were quenched with apyrase^59^. FRET signals of the E2~Ub conjugates were recorded as a function of time following addition of different RING constructs using AF488 excitation (485/20 nm) and AF594 emission (645/40 nm) bandpass filters. We observed that more than 40% of the total fluorescence signal decayed in a RING-dependent fashion and could, therefore, be attributed to the FRET signal of the conjugate. The outstanding sensitivity, signal-to-noise, and throughput of the FRET decay measurements enabled robust and accurate quantification of ubiquitin discharge.

We first investigated ubiquitin discharge from the UBE2N~Ub conjugate in the heterodimer with UBE2V2 (UBE2N~Ub/V2) catalyzed by different TRIM5α RING constructs (Fig. 3b). In general, reactions catalyzed by E3 ubiquitin ligases are bisubstrate reactions, in which ubiquitin is transferred from a E2~Ub conjugate onto a substrate protein. The UBE2N/V2 heterodimer is a specialized E2 that transfers ubiquitin of the UBE2N~Ub conjugate (donor ubiquitin) onto lysine 63 of another ubiquitin (substrate or acceptor ubiquitin). The UBE2N~Ub/V2 FRET ubiquitin discharge assays were performed in the presence of 10 μM of free ubiquitin that acted as the ubiquitylation substrate. Even though ubiquitin discharge is a bisubstrate reaction, Michaelis-Menten analysis can be used to determine the apparent k_cat_/K_m_ for the E2~Ub substrate when the concentration of the second substrate (10 μM of free ubiquitin) is kept constant (see Supplementary Methods). The ubiquitin discharge rates were measured as a function of the enzyme concentration, instead of the substrate (E2~Ub) concentration, as is explained in more detail in Supplementary Methods. The observed ubiquitin discharge rates were plotted as a function of RING concentration, and the initial slopes of these plots (the apparent k_cat_/K_m_) were used to quantify the E3 activity of each RING construct (Fig. 3d). In agreement with previous work^46^, we find that catalysis of ubiquitin discharge from the UBE2N~Ub/V2 conjugate is virtually abolished by the I77R mutation in the R1-I77R and R3-I77R constructs. The E3 activity of the R2-WT is also significantly higher than that of the R1-WT. These observations confirm that RING dimerization promotes ubiquitin discharge from the UBE2N~Ub/V2 conjugate by stabilizing a particular relative arrangement of UBE2N and donor ubiquitin, in which the high-energy thioester bond is exposed and primed for nucleophilic attack by the K63 amine of the substrate ubiquitin. We also observe that the E3 activity per RING domain is lower in the R3-WT construct than in R2-WT. This observation is in agreement with our NMR data, which indicate that in the R3-WT sample a portion of RING domains remain in the monomer-like, catalytically inactive conformation.

We then used the FRET assay to investigate RING-catalyzed ubiquitin discharge from UBE2W, another specialized E2 known to mediate the antiviral activity of TRIM5α^37^. UBE2W displays specificity for the N-terminal amines in flexible N-terminal segments of proteins as ubiquitylation substrates^60,61^. Because the FRET ubiquitin discharge reactions described here were performed in a complex mixture of many different proteins there is some uncertainty regarding the substrate of ubiquitin discharge from the UBE2W~Ub conjugate. The issue of the ubiquitylation substrate will be addressed in the next section. Here, as in the UBE2N/V2 experiments described above, ubiquitin discharge was investigated as a function of RING concentration, whereas concentrations of all other reaction components were kept constant. Kinetics of FRET decay observed in the UBE2W experiments was notably different from what was observed for UBE2N/V2. The initial rapid burst of FRET decay was followed by a slower decay phase, the amplitude of which, but not the rate, was dependent on the RING concentration (Fig. 3c). The data reveal that this bisubstrate reaction is not well described by the Michaelis-Menten model and the mechanistic basis of this distinctive kinetics will require further study. However, the dataset offers another illustration of the power of the FRET-based ubiquitin discharge assay for studies of E3 ubiquitin ligases and allows quantitative comparison of the enzymatic activity of different constructs. Because the rate of the initial burst, albeit RING concentration-dependent, was too fast to be measured accurately, the amplitude of the RING-dependent FRET decay was used instead (Supplementary Fig. 2C). Dependence of decay amplitude on RING concentration displayed a good fit to the Michaelis-Menten equation, and the initial slope (analog of k_cat_/K_m_) was used as a quantitative measure of the E3 activity. This dataset revealed that albeit the discharge from the UBE2W~Ub conjugate was less sensitive to the I77R mutation, the R3-I77R and R1-I77R constructs retained less than 10% of the R3-WT and R1-WT E3 activity, respectively. These data establish that akin to UBE2D^49–51^ and UBE2N/V2^46–48,62^, UBE2W is another E2 whose ability to transfer ubiquitin onto its substrates is strongly enhanced by RING dimerization. Another notable difference with the UBE2N/V2 dataset was the significantly higher activity of the R3-WT construct in comparison to R2-WT. We hypothesized that this enhancement resulted from the third RING in the R3-WT construct acting as the substrate for ubiquitin transfer^37,38^ and proceeded to a more careful examination of the products of the ubiquitylation reactions catalyzed by our engineered RING constructs.

### Proximity of the third RING with an unstructured N terminus facilitates N-terminal monoubiquitylation (priming) of TRIM5α by UBE2W and subsequent extension of the TRIM5α-attached K63-linked polyubiquitin chain by UBE2N/V2

**Fig. 4:**
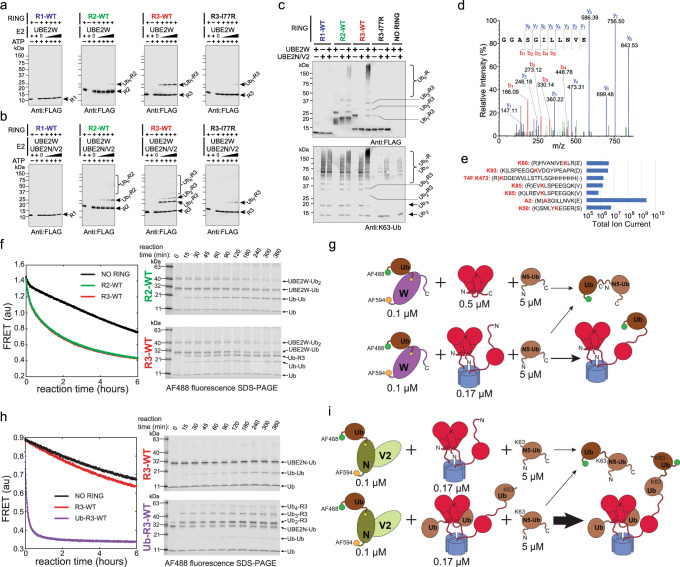
Proximity of three RING domains promotes each of the two distinct steps in the autoubiquitylation of TRIM5α: the N-terminal monoubiquitylation (priming) and the extension of the TRIM5α-anchored K63-linked polyubiquitin chain. **a**, **b** RING autoubiquitylation products visualized by WB of FLAG-tagged RING constructs. [RING] = 5 µM, [Ub] = 10 µM, [E2] = 0.1; 0.2; 0.5; 1 µM. 30 min incubation. **a** Monoubiquitylation of RING in the presence of the increasing amounts of UBE2W. **b** RING polyubiquitylation in the presence of both UBE2W and the heterodimeric UBE2N/V2. **c** Ubiquitylation products of different E2/E3 combinations analyzed side-by-side on the same SDS-PAGE gel. [RING] = 5 µM, [Ub] = 10 µM, [E2] = 1 µM. 30 min incubation. WB was performed with either anti-FLAG antibodies (top panel) or anti-K63-linked-Ub antibodies (bottom panel). **d** N-terminal anchoring of ubiquitin in the R3 WT construct is confirmed by mass spectrometry. **e** N-terminal monoubiquitylation is preferred over other modification sites by more than two orders of magnitude. **f** Kinetics and products of ubiquitin discharge from the AF594-UBE2W~AF488-Ub conjugate (0.1 μM) catalyzed by R2-WT ([RING] = 1 μM; [dimer] = 0.5 μM) or R3-WT constructs ([RING] = 0.5 μM; [trimer] = 0.17 μM) in the presence of [N5-Ub] = 5 μM. **g** Cartoon representation of the UBE2W reactions and the corresponding products. **h** Kinetics and products of ubiquitin discharge from the AF594-UBE2N~AF488-Ub/UBE2V2 conjugate (0.1 μM) catalyzed by R3-WT ([RING] = 0.5 μM; [trimer] = 0.17 μM) or Ub-R3-WT constructs ([RING] = 0.5 μM; [trimer] = 0.17 μM) in the presence of [N5-Ub] = μM. **i** Cartoon representation of the UBE2N/V2 reactions and the corresponding products.

We first investigated ubiquitylation products generated by the RING constructs using Western blot (Fig. 4a, b). A C-terminal FLAG tag was fused to the RING constructs and used to visualize products formed in conventional in vitro ubiquitylation reactions containing E1, different E2 combinations, ubiquitin, and other required components. UBE2W displays specificity for unstructured N-terminal segments in substrate proteins^60,61^ and the reaction usually stops after addition of a single ubiquitin to the N-terminal amine of the substrate because ubiquitin lacks an unstructured N-terminal tail and is a poor substrate for UBE2W. In agreement with previous work^37,38^, when UBE2W was the only E2 included in the reaction, a single additional band corresponding to monoubiquitylated RING construct was the predominant product of the ubiquitylation reaction (Fig. 4a). Although some RING monoubiquitylation could be observed in all reactions, modification of the R3-WT construct was clearly the most efficient. When UBE2N and UBE2V2 are included in these ubiquitylation reactions together with UBE2W, we observe polyubiquitylation of the TRIM5α RING constructs and once again the R3-WT is the most active among the tested constructs (Fig. 4b).

For a more direct and accurate comparison of products formed in different combinations of UBE2W, UBE2N/V2 and various RING constructs we performed all reactions in parallel and analyzed the products on a single SDS-PAGE gel by Western blotting with either anti-FLAG or anti-K63-linked-polyubiquitin antibodies (Fig. 4c). The anti-FLAG WB analysis confirmed that the R3-WT construct displayed most potent autoubiquitylation activity. It also revealed that RING polyubiquitylation by UBE2N/V2 requires prior monoubiquitylation by UBE2W, in agreement with prior studies using different TRIM5α constructs^37,38^. Another important effect of UBE2W-mediated monoubiquitylation on UBE2N/V2-dependent products is evident from the WB with anti-K63 antibodies. UBE2N/V2 in known to have some background activity that results in the synthesis of unconjugated, K63-linked polyubiquitin chains in vitro, and we can detect this activity in our experiments. We observe that synthesis of unconjugated K63-linked polyubiquitin is strongly enhanced by all dimerization-competent RING constructs, R1-WT, R2-WT, and R3-WT, but not by the R3-I77R mutant. Whereas inclusion of UBE2W had only a minor effect on the synthesis of unconjugated K63-linked polyubiquitin by the R1-WT and R2-WT constructs, the R3-WT construct displayed a notably different pattern of low molecular weight products and significantly denser staining at high molecular weights that overlapped with the anti-FLAG staining of polyubiquitylated R3-WT. These observations revealed that once the R3-WT trimer is monoubiquitylated by UBE2W a much larger fraction of total K63-linked polyubiquitin products generated by UBE2N/V2 is covalently attached to the RING rather than unconjugated.

Series of in vitro and cellular experiments by Fletcher et al. strongly indicated that the N-terminus is the functionally relevant modification site of TRIM5α by UBE2W^38^. We used mass spectrometry (MS) to verify that the robust UBE2W-mediated autoubiquitylation of the R3-WT construct similarly favors the N-terminal attachment of ubiquitin. Indeed, the MS analysis revealed that modification of the N-terminal amine in R3-WT by UBE2W was preferred over other modification sites by more than two orders of magnitude as indicated by the corresponding ion currents (Fig. 4d, e).

We then asked whether the asymmetry of the trimeric R3-WT construct results in the asymmetry of the formed products, with only one out of three RINGs within the trimer modified with ubiquitin. To this end we labeled the R3-WT construct with a C-terminal fluorescent tag using sortase (Supplementary Fig. 2A) and quantified monoubiquitylation stoichiometry as a function of time by directly imaging and integrating fluorescence intensity of the R3-WT and Ub-R3-WT bands in SDS-PAGE gels (Supplementary Fig. 3A). This fluorescent SDS-PAGE analysis is immune to non-linearities associated with membrane transfer and antibody staining in WB. We observed that monoubiquitylation of the R3-WT construct goes to completion with no apparent accumulation of intermediates at 1:3 stoichiometry. These observations suggest that the monomer-dimer exchange within the R3-WT construct enables monoubiquitylation of all three RINGs by UBE2W.

Collectively, results in Fig. 4a–e confirm that our engineered RING constructs recapitulate the activity and the products generated by the full-length TRIM5 in cell-based experiments^37,38^. Furthermore, the distinctive catalytic properties of the R3-WT construct indicate that proximity of three, rather than two, RING domains is functionally significant. To evaluate whether the proximity of three RINGs enhances the rate of inter-trimer RING autoubiquitylation compared to the rate of intermolecular reactions we once again turned to our FRET assays. To minimize the number of assay components, we used purified FRET-active E2~Ub conjugates in this set of ubiquitin transfer reactions. As we explain in the previous section, the K63 residue of ubiquitin is the preferred substrate of the UBE2N~Ub/V2 conjugate, but the N-terminal amine of ubiquitin is not a good substrate for UBE2W~Ub because ubiquitin lacks a flexible N-terminus. To provide a good alternative substrate for both, UBE2N/V2-mediated and UBE2W-mediated ubiquitylation, we used an engineered ubiquitin, which contained a 5-residue (GGGGS) N-terminal extension preceding the starting methionine of the WT ubiquitin (N5-Ub). Different RING constructs and 5 μM of the N5-Ub substrate were added to the UBE2W~Ub or UBE2N~Ub/V2 conjugates and the FRET signal was used to monitor ubiquitin discharge from the conjugate. In addition, series of time point samples were collected for the analysis of the products by SDS-PAGE imaged using AF488 fluorescence.

First, we compared ubiquitin transfer from the UBE2W~Ub conjugate catalyzed by the R2-WT and R3-WT constructs (Fig. 4f, g). Structures of the RING dimer complexes with E2~Ub conjugates reveal that the same RING dimer is unlikely to act as both the catalytic activator and the substrate in ubiquitin transfer. In agreement, the main product observed in the reaction of 0.1 μM UBE2W~Ub, 0.5 μM R2-WT, and 5 μM N5-Ub mixture is di-ubiquitin (Ub-Ub) formed in an “intermolecular” transfer of AF488-Ub from UBE2W~Ub onto the N5-Ub substrate. In contrast, in the reaction of 0.1 μM UBE2W~Ub, 0.5 μM R3-WT, and 5 μM N5-Ub, the dominant product is the monoubiquitylated Ub-R3-WT despite the 30-fold excess of N5-Ub over RING trimer. These observations strongly support the “intramolecular” mechanism of R3-WT monoubiquitylation, in which the catalytic RING dimer and the substrate RING reside within the same R3-WT trimer.

We then explored how proximity of three RINGs affects the rates of ubiquitin transfer reactions mediated by UBE2N/V2 (Fig. 4h, i). To that end we prepared a Ub-R3-WT construct, in which the full-length WT Ub sequence is cloned immediately preceding the residue A2 of the RING, yielding the fusion protein that is chemically identical to the product of N-terminal monoubiquitylation of R3-WT by the UBE2W. Once again, the dominant product of the reaction containing 0.1 μM UBE2N~Ub/V2, 0.5 μM R3-WT, and 5 μM N5-Ub was di-ubiquitin (Ub-Ub) as expected for the intermolecular transfer of 488-Ub from UBE2N~Ub onto K63 of N5-Ub. Notably, when the reaction is performed with Ub-R3-WT instead of R3-WT, the rate of FRET decay is dramatically enhanced, and the RING-conjugated products (Ub_2_-R3-WT and Ub_n_-R3-WT) are greatly favored. The striking increase of the apparent ubiquitin transfer rate reveals that monoubiquitylated RING trimer is an exceptionally good substrate for ubiquitin chain extension by UBE2N~Ub/V2 owing to the proximity of the monoubiquitylated substrate RING monomer to the catalytic RING dimer in the Ub-R3-WT construct.

Finally, we also carried out the UBE2N/V2 experiments with the Ub-R3-WT trimer that was prepared by first performing the enzymatic monoubiquitylation of the R3-WT construct using UBA1 and UBE2W and then by purifying the ubiquitylated RING products (Supplementary Fig. 3B–E). In the enzymatically monoubiquitylated Ub-R3-WT the monoubiquitylation did not go to completion and approximately half of the total RING domains were monoubiquitylated in the purified Ub-R3-WT sample (Supplementary Fig. 3B). Experiments performed with this sample also revealed a major enhancement of the ubiquitin transfer rate and a shift in ubiquitylation products (Supplementary Fig. 3D, E), in agreement with the results obtained with the genetically ubiquitylated trimer. Collectively, the data in Fig. 4 and Supplementary Fig. 3 establish that proximity of three RINGs accelerates the rates of the priming and extension reactions in the autoubiquitylation of TRIM5α.

## Discussion

E3 ubiquitin ligases from the TRIM family are increasingly recognized for their diverse contributions to innate immune defenses^4^, and the biochemical mechanisms that control polyubiquitin synthesis by TRIM proteins are of great interest. For example, TRIM5α-mediated restriction of retroviral replication depends on direct association of TRIM5α with the retroviral capsid, but the capsid is not the ubiquitylation substrate of this E3 ligase. Instead, TRIM5α is thought to undergo autoubiquitylation upon binding to the capsid, and here we investigate how association of TRIM5α with the capsid promotes its autoubiquitylation.

We show that the mechanism of TRIM5α E3 activation depends on proximity of three RING domains. At the vertices of the honeycomb-like assemblies formed by TRIM5α on the capsid surface, three RING domains from three different TRIM5α dimers are brought together by the interactions mediated by the B-box domains^10^. Proximity of three RINGs leads to an asymmetric arrangement in which two RING domains associate into a compact dimer, whereas the third RING remains in a monomer-like state. The asymmetry of this arrangement arises from the disorder-to-order transitions in the polypeptide segments flanking the Zn-binding core of the RING domain, which are largely unstructured and mobile in the RING monomer, but fold into a well-defined amphipathic four-helix bundle in the dimer. The findings elucidate how the symmetry mismatch between B-box trimerization and RING dimerization enables the dual catalytic and substrate functionality of the RING domain and activates TRIM5α autoubiquitylation upon capsid binding.

**Fig. 5:**
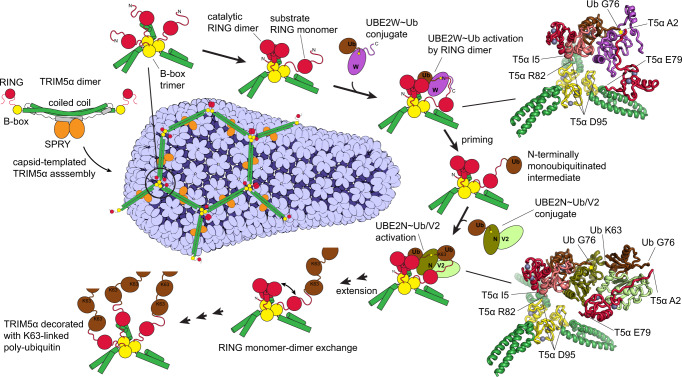
Transient proximity of three RING domains promoted by the association of TRIM5α with the retroviral capsid explains how TRIM5α autoubiquitylation is activated by capsid binding. The honeycomb-like TRIM5α assembly on the surface of the retroviral capsid is required for the antiviral activity of the protein (see a recent comprehensive review for detailed bibliography^10^). The architecture of the TRIM5α assembly suggests that the cooperativity between SPRY:capsid interactions and B-box:B-box interactions forms the basis of the pattern recognition functionality and promotes capsid binding^22^. B-box trimerization at the vertices of the TRIM5α honeycomb brings three N-terminal RING domains into proximity. Here we show that proximity of three RINGs creates an asymmetric structural arrangement, which explains how RING can act as both the catalytic activator and the substrate in the ubiquitin transfer reactions. Fluorescent ubiquitin discharge assays reveal that proximity of three RINGs strongly enhances the rates of the two distinct ubiquitin transfer processes involved in the autoubiquitylation of TRIM5α: the N-terminal priming of TRIM5α with a single ubiquitin by UBE2W and the subsequent extension of the TRIM5α-attached K63-linked polyubiquitin chain by UBE2N/V2. The findings imply that both the priming and the extension are enhanced by the association with the capsid. Relative arrangement of protein subunits in the complexes mediating these events can be modeled based on extensive existing structural data (see “Methods”)^26, 34, 46–50, 53–56, 63^. The models (insets in the upper right and lower right corners of the figure) illustrate how proximity of the third RING and the flexibility of its backbone facilitate ubiquitin transfer in the two complexes. Collectively, the findings elucidate how the symmetry mismatch between B-box trimerization and RING dimerization enables the dual activator and substrate functionality of the RING and activates TRIM5α autoubiquitylation upon capsid binding.

The wealth of structural data on different TRIM5α domains, TRIM5α-capsid interactions and RING-mediated recruitment and activation of E2~Ub conjugates makes it possible to build structural models of the protein complexes that mediate autoubiquitylation of TRIM5α (Fig. 5)^26,34,46–50,53–56,63^. This study reveals how the disorder-to-order transitions and the asymmetry of the RING trimer enable the distinct functional contributions of the three RINGs to the autoubiquitylation process. The RING dimer performs a catalytic role by stabilizing a particular relative orientation of E2 and ubiquitin in the E2~Ub conjugate, in which the thioester bond is exposed and primed for ubiquitin transfer. Here we show that this mechanism contributes to the activation of the two E2s known to mediate the antiviral function of TRIM5α: UBE2W and the heterodimeric UBE2N/V2 (Fig. 3). Structural constraints prevent RING dimers from catalyzing transfer of the ubiquitin onto itself because the thioester bond in the active site of the E2~Ub conjugate points away from the E2-RING interface. The asymmetry of the RING trimer therefore reflects the inherent functional asymmetry in the process of RING autoubiquitylation, in which two RINGs perform a catalytic role and the third RING acts as the ubiquitylation substrate.

The proximity of the third RING and the mobility of its backbone are likely to facilitate the autoubiquitylation process in several ways. Long and mobile N-termini in proteins are known to be the preferred ubiquitylation substrates for UBE2W^60^. In addition, the backbone flexibility may further enhance autoubiquitylation by extending the reach of the third RING and increasing the rate at which its N-terminus encounters the active site of the UBE2W~Ub conjugate recruited and activated by the RING dimer. Proximity and backbone flexibility elevate the effective concentration of the preferred substrate in the vicinity of the activated UBE2W~Ub conjugate and make the N-terminus of the third RING the predominant ubiquitylation site that outcompetes other potential substrates (Fig. 4f, g). Our findings explain why the engineered human TRIM5α variant containing two tandem RING domains can activate NF-κB only when the I76R mutation is introduced into one of the two RINGs^38^. Once the third RING is monoubiquitylated, the same mechanism greatly favors the K63 residue of the N-terminally attached ubiquitin as the preferred site for ubiquitin transfer from the UBE2N~Ub/V2 conjugate. This explains the strong enhancement of the ubiquitin discharge rate and the change in the products of UBE2N/V2-mediated polyubiquitin synthesis that we observe with the monoubiquitylated trimeric Ub-R3-WT construct (Fig. 4h, i). The question whether the unconjugated or conjugated K63-linked polyubiquitin is the functionally important signaling species has been controversial in the studies of RIG-I signaling^6^. Our findings indicate that the TRIM5α-attached, K63-linked polyubiquitin (rather than unconjugated polyubiquitin chains) is the functionally important product that mediates downstream events promoted by the E3 activity of TRIM5α assembled on the capsid surface. In addition, we present evidence that RING monomers can swap their roles within the RING trimer (Supplementary Fig. 3A), which suggests that all three RINGs can eventually be modified and that the vertices of the honeycomb-like TRIM5α assemblies can become decorated with up to three polyubiquitin chains.

Finally, the requirement for proximity of three RING domains as the minimum number needed for efficient autoubiquitylation offers insight into how the E3 activity of TRIM protein can be activated by three-dimensional patterns of binding epitopes. Self-association of isolated TRIM5α B-box domains in vitro is known to be relatively weak and the assembly of full-length TRIM5α dimers on the capsid surface is thought to be enhanced by the avidity effect arising from the interaction of the SPRY domains with multiple distinct binding epitopes on the capsid surface^26,64^. The cooperativity between B-box association and SPRY-capsid interactions is expected to be strongly dependent on the distance between SPRY binding epitopes, as dictated by the length of the coiled-coil rods, and the angles between them, as dictated by the preferred geometry of B-box self-association^10^. The reversible self-association of the mobile RING segments into a four-helix bundle of the RING dimer described here is likely to provide an additional contribution to the capsid binding avidity and the cooperativity of the TRIM5α assembly/disassembly process. As a result, proximity of three RING domains depends on binding of at least three TRIM5α dimers to a particular spatial arrangement of three distinct and distant sets of epitopes recognized by the SPRY domains^22^. Furthermore, because the capsid-templated TRIM5α assembly/disassembly process is highly cooperative, formation of a larger honeycomb segment may be required to ensure that RING trimers persist long enough to complete synthesis of multiple polyubiquitin chains at the vertices of the honeycomb. In summary, the cooperativity between ligand binding mediated by the C-terminal domains and self-association mediated by the B-box and RING domains of TRIM proteins ensures that foci of K63-linked polyubiquitin chains are only synthesized when multiple TRIM binding epitopes are arranged in a particular pattern in three dimensions.

Conserved domain architecture of the TRIM family suggests that the mechanism that links transient proximity of multiple RING domains to E3 activation is conserved in one form or another across the TRIM family. Indeed, structural and functional studies on TRIM21 and TRIM25 revealed numerous functional similarities between these TRIM family members suggesting that the mechanisms controlling their E3 activity are closely related^40,47,48,52–54^. Although the exact role of K63-linked polyubiquitin in inducing antiviral immune responses is not fully understood, K63-linked polyubiquitin chains are thought to promote assembly and activation of various multiprotein complexes mediating downstream immune events^65^. Collectively, results reported here and in other recent studies on TRIM5α and related E3 ligases shed light on the ability of TRIM proteins to detect particular patterns of multiple binding epitopes and to create immunostimulatory foci of K63-linked polyubiquitin either through autoubiquitylation, or through ubiquitylation of other substrates.

## Methods

### Protein expression and purification

All recombinant proteins used in the study were expressed in bacteria and purified using standard protocols (Supplementary Table 1). All protein modifications (mutations, insertions, and deletions) were performed using the QuickChange Lightning Multi Site-Directed Mutagenesis Kit (Agilent Cat # 200513). Isotopic labeling of proteins for NMR studies was achieved by culturing bacteria in isotopically enriched minimal media (M9) following standard procedures. The N-terminal methionine (M1) residue is post-translationally removed from TRIM5α in mammalian cells^37,38^, we therefore used alanine 2 (A2) as the starting residue in the fusion RING constructs. The R1-WT and R1-I77R constructs encompass residues 2–90 of rhesus monkey TRIM5α. The R2-WT tandem construct had the following residue composition: 2-82-Gly-Ser-2-92. The R3-WT construct contained residues 1–95 fused on the C-terminus to the T4 fibritin foldon peptide sequence followed by a 6His tag. R1-WT and R2-WT were cloned into custom pET30a expression vectors as fusions containing a 6His tag, followed by a GB1 solubility tag, followed by a TEV cleavage site at the N-terminus. The tags were removed by TEV protease digestion using standard protocols. The R3-WT construct was cloned into a pET11a vector. The start-codon N-formylmethionine in the R3-WT construct was naturally removed by the bacterial methionine aminopeptidase. Expression and purification of E2s and ubiquitin was performed using previously published procedures^59^. Briefly, bacterial cells were grown in LB media to an OD_600_ ~ 0.6 at 37 °C. Protein expression was induced by adding 1 mM isopropyl-β-D-thiogalactopyranoside followed by overnight incubation at 20 °C. Cells were disrupted by sonication and the cell lysate was centrifuged at ~40,000 × *g* for 60 min. The supernatant was loaded onto a Ni-NTA column and the target proteins were subsequently eluted with 0.5 M imidazole. The eluted proteins were further purified on a Superdex 75 column (GE Healthcare) containing 50 mM Tris-HCl pH 8, 100 mM NaCl, and 1 mM TCEP. Sortase recognition peptides or FLAG tags were added to some constructs as described in text and in Supplementary Fig. 2A, B.

### NMR spectroscopy

All NMR experiments were performed in 50 mM sodium phosphate, pH 7.5, 150 mM NaCl, 1 mM TCEP, 10% (v/v) D_2_O, 0.04% (w/v) NaN_3_, and the data collected at 298 K on a Bruker Avance 700-MHz spectrometer equipped with a cryoprobe using Bruker TopSpin v3.2. Backbone assignments were carried out using standard TROSY triple resonance NMR experiments (HNCO, HN(CA)CO, HN(CO)CA, HNCA, HNCACB, and HN(CO)CACB). Protein concentrations for each experiment are shown in the corresponding figures or noted in figure legends. The NMR data were processed using NMRPipe v8.7 and spectra evaluated using NMRFAM-Sparky 1.413.

### Analytical ultracentrifugation

Sedimentation velocity experiments were performed with a Beckman Optima XL-I analytical ultracentrifuge (Beckman Coulter, Indianapolis, IN) equipped with a 4-hole AnTi-60 rotor and 2-channel centerpiece cells. Samples were in a buffer containing 50 mM Tris-HCl, pH 7.5, 150 mM NaCl, and 1 mM TCEP. Sedimentation velocity experiments were carried out at 20 °C and data collection performed in the intensity mode at 280 nm using Proteomelab XLI AUC v6.2 software. Sedimentation velocity datasets were analyzed with SEDFIT 16.1 using continuous size distribution c(S) model. All c(S) distributions were calculated with fitted frictional ratios (f/f_0_) and meniscus positions were all fitted with a maximum entropy regularization cutoff of 0.95. Sedimentation equilibrium experiments were carried out using six-channel centerpieces, each chamber containing 110 μL samples. Equilibrium experiments were conducted at 20 °C and collection of data performed in the intensity mode at 280 nm and 230 nm. A total of 60 scans were collected at each equilibrium speed and analyzed using SEDFIT 16.1 to determine when equilibrium was reached. Final scans for each sample and speeds were analyzed using SEDPHAT 15.2b. Equilibrium distributions were fitted to a monomer-dimer self-association model.

### Sortase-catalyzed fluorescent labeling

Fluorescent labeling of proteins was carried out by sortase-catalyzed transpeptidation^58^. Briefly, for N-terminal labeling of E2s and ubiquitin the proteins were expressed with N-terminal affinity tags followed by a TEV protease cleavage site. Treatment with TEV protease was used to remove the fusion tag leaving an N-terminal glycine residue on the cleaved protein. Short fluorescent peptides used for the N-terminal sortase-catalyzed labeling contained a fluorescent dye at the N terminus and a sortase recognition sequence (KLPETGG) at the C terminus. Labeling reactions were carried out using 80 μM fluorescently labeled peptide, 20 μM sortase and 20 μM of the target protein for 30 min at 37 °C. Unreacted fluorescent peptides were separated from the labeled proteins by size exclusion chromatography. C-terminal labeling of the R3-WT construct was achieved by adding a sortase recognition sequence (KLPETGG) at the C terminus of the construct and using a short fluorescent peptide containing GGGG sequence at the N terminus and the AF594 fluorescent dye at the C terminus.

### Ubiquitylation assays

Unless specified otherwise, ubiquitylation reactions were performed in 100 μL volumes and contained 1 mg/mL of E1, 0.1–1 μM E2, 1 μM E3, 50 μM Ub, and 5 mM ATP in the reaction buffer (50 mM Tris-HCl, pH 7.4, 150 mM NaCl, 5 mM MgCl_2_, 1 mM DTT, 1%(v/v) glycerol, 3 U/mL creatine phosphokinase, and 5 mM creatine phosphate). For reactions, containing fluorescent Ub, reactions were supplemented with 5 μM fluorescent Ub. Reactions were incubated at 37 °C for 1 h. Reactions were quenched with the addition of reducing SDS-PAGE buffer and loaded onto SDS-PAGE gels (Invitrogen Cat# XP102005BOX). Following SDS-PAGE, gels were either imaged using a Typhoon TRIO imager v5.0 or transferred onto nitrocellulose membranes for Western blotting.

For Western immunoblotting, membranes were probed using anti-Ub (1:1000—CST Cat # 3933S), anti-K63 Ub (1:1000—CST Cat #3496S), or anti-FLAG (1:5000—Millipore Cat #F1804). Secondary fluorescent antibodies (1:10,000—LICOR Cat # 926–3211 and 926–68073) were used to detect Ub and RING products using a LI-COR Odyssey scanner. Western blot analysis was performed using ImageStudio Lite (Ver 5.2).

### Mass spectrometry

TRIM5 ubiquitylation site was determined by incubating R3-WT (100 μM) with UBE2W (5 μM) and ubiquitin (125 μM) in ubiquitylation reaction buffer (see above). Reaction was run for 16 hr at room temperature, Ub-R3-WT was then isolated via metal chelate affinity chromatography (Ni-NTA), and then by size-exclusion chromatography on a Superdex-75 column (GE Life Sciences Cat # 29-1487-21) (Supplementary Fig. 3B). Purified Ub-R3-WT was run on SDS-PAGE, the Ub-R3-WT band was excised, reduced/alkylated and digested with trypsin. The peptides were analyzed by LC-data-dependent acquisition tandem mass spectrometry on an Orbitrap Velos Pro (Thermo Fisher) with data processing by Mascot (Matrix Science) and Scaffold (Proteome Software).

### FRET ubiquitin discharge assays

Fluorescently labeled E2~Ub conjugates for FRET-based ubiquitin discharge assays were prepared by incubating 2.5 mg/mL E1, 5 μM AF594-labeled E2, 5.5 μM AF488-labeled Ub, and 5 mM ATP in conjugation buffer (50 mM Tris-HCl, pH 7, 150 mM NaCl, 5 mM MgCl_2_, 1 mm DTT, 1% (v/v) glycerol, 0.5 mg/mL BSA) for 30 min at 37 °C. The reactions were then quenched by depleting ATP with 2 U/mL of apyrase (New England BioLabs Cat# M0398S) for 5 min at room temperature. The reactions were then diluted and dispensed into 384-well fluorescence microplate to yield 125 nM final concentration of the fluorescent E2~Ub conjugate. Discharge reactions were run in discharge buffer (50 mM Tris-HCl, pH 7, 150 mM NaCl, 5 mM MgCl_2_, 1% (v/v) glycerol, 0.5 mg/mL BSA, 10 μM free unlabeled ubiquitin) at room temperature by adding different amounts of RING constructs to the fluorescent E2~Ub conjugates. Fluorescence intensity measurements were performed in 384-well plates on a Synergy 2 microplate reader (BioTek). All experiments were performed in duplicate with 70-μL sample volumes in each well. Fluorescence decay curves were analyzed using MATLAB (R2019b) as described in text, in Supplementary Methods and in Supplementary Fig. 2C. Purified FRET-active E2-Ub conjugates are now commercially available from E3 Bioscience LLC (e3bioscience.com).

### Circular dichroism spectropolarimetry

CD experiments were performed on a Jasco J-815 spectropolarimeter, equipped with a PTC-423S Peltier temperature control unit, using a cuvette with 1 mm path length. Varying concentrations of RING monomer or dimer were dissolved in 50 mM sodium phosphate 7.4, 100 mM NaCl, and 1 mM TCEP. The CD spectra were recorded at 25 °C and the experimental ellipticity values at 222 nm were used to calculate molar ellipticity for each sample using the following formula: Molar ellipticity = m°/(10*L*C); where m° is raw sample ellipticity in millidegrees, C is RING domain concentration in each sample (e.g., in the 20 µM sample of R2-WT, [RING] = 40 µM) and L is path length of cell (cm). Molar ellipticities were then plotted as a function of RING domain concentration in each sample in Fig. 2c.

### Complex illustration and modeling

Protein complexes that mediate the priming and extension of the TRIM5α-conjugated polyubiquitin chains can be modeled based on extensive existing structural data (insets in Fig. 5). Three RING domains are held in proximity by the B-box trimer^26^. Two out of three RINGs associate into a dimer whose crystal structure is known and similar to other known TRIM RING dimers^46–48,54–56^. In the third RING, the segments that form the four-helix bundle in the dimer are largely unstructured as observed in the NMR structure of the human TRIM5α RING^34^. The catalytic RING dimer and E2s form a canonical interface observed in the crystal structure of the TRIM5α RING:UBE2N complex^46^ and in highly similar complexes of UBE2N with RINGs from TRIM25 and TRIM21^47,48,53,54^. TRIM5α RING:UBE2W interface can be modeled using RING:UBE2N structures. Activation of the donor ubiquitin of the E2~Ub conjugate mediated by the interaction with the catalytic RING dimer is conserved and can be modeled using numerous known structures of RING:E2~Ub complexes^47–50,53,54^. Positioning of the acceptor ubiquitin by the UBE2N/V2 complex that forms basis for K63 specificity is also well established^53,63^. The structural basis of the UBE2W specificity for the N-terminal amine remains unknown and cannot be modeled. The model of the UBE2W complex only illustrates that mobility of the N-terminal and C-terminal backbone segments helps the substrate RING span the distance between the B-box trimer and the active site (thioester bond) of the UBE2W~Ub conjugate.

### Reporting summary

Further information on research design is available in the Nature Portfolio Reporting Summary linked to this article.

## Supplementary information

Supplementary Information

Reporting Summary

## Data Availability

The crystal structures used in this study are obtained from the Protein Data Bank PDB IDs 2ECV, 4TKP, and 5IEA. Data supporting the findings of this paper are available from the corresponding authors upon request. Source data are provided with this paper.

## Source data

Source Data
