# Supplementary material for: Structural and functional asymmetry of RING trimerization controls priming and extension events in TRIM5α autoubiquitylation
Source: Nat Commun. 2022 Nov 19;13:7104. doi: 10.1038/s41467-022-34920-3 (PMC9675739; doi:10.1038/s41467-022-34920-3)
Supplement: Supplementary file 1 — Supplementary Information [file 41467_2022_34920_MOESM1_ESM.pdf]

SUPPLEMENTARY INFORMATION

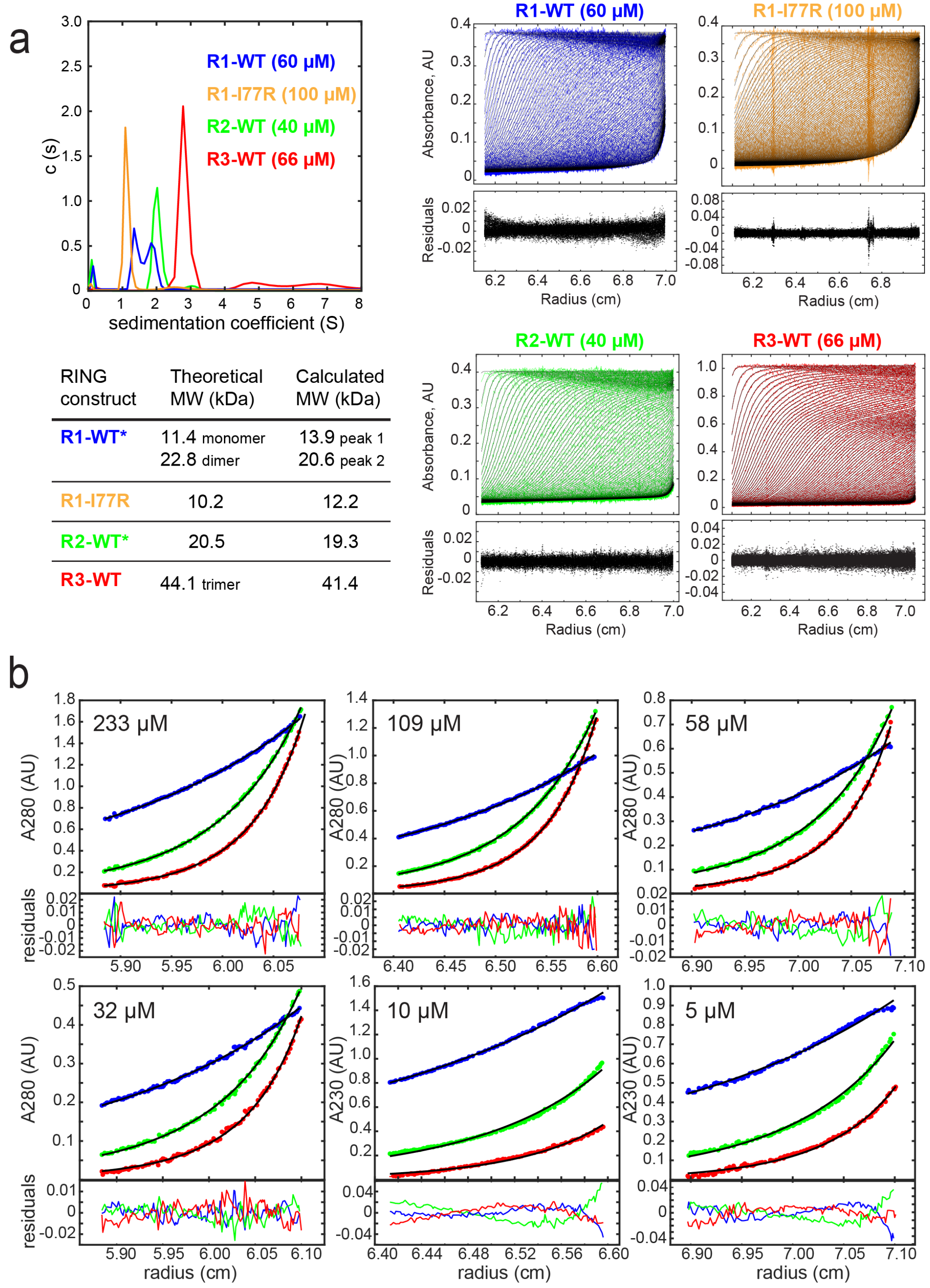

RING construct	Theoretical MW (kDa)	Calculated MW (kDa)
R1-WT*	11.4 monomer<br>22.8 dimer	13.9 peak 1<br>20.6 peak 2
R1-I77R	10.2	12.2
R2-WT*	20.5	19.3
R3-WT	44.1 trimer	41.4

Supplementary Figure 1. Analytical ultracentrifugation studies of RING constructs. (A) Sedimentation velocity analysis of RING constructs. Constructs denoted with asterisk (*) contained a C-terminal FLAG tag. (B) RING dimerization investigated by the sedimentation equilibrium analysis of the R1 WT construct. Equilibrium distributions measured for different RING concentrations at 19000 rpm (blue), 26000 rpm (green), and 36000 rpm (red) were fitted to a monomer-dimer self-association model yielding a K_D = 63 ± 28μM. Source data are provided as a Source Data File.

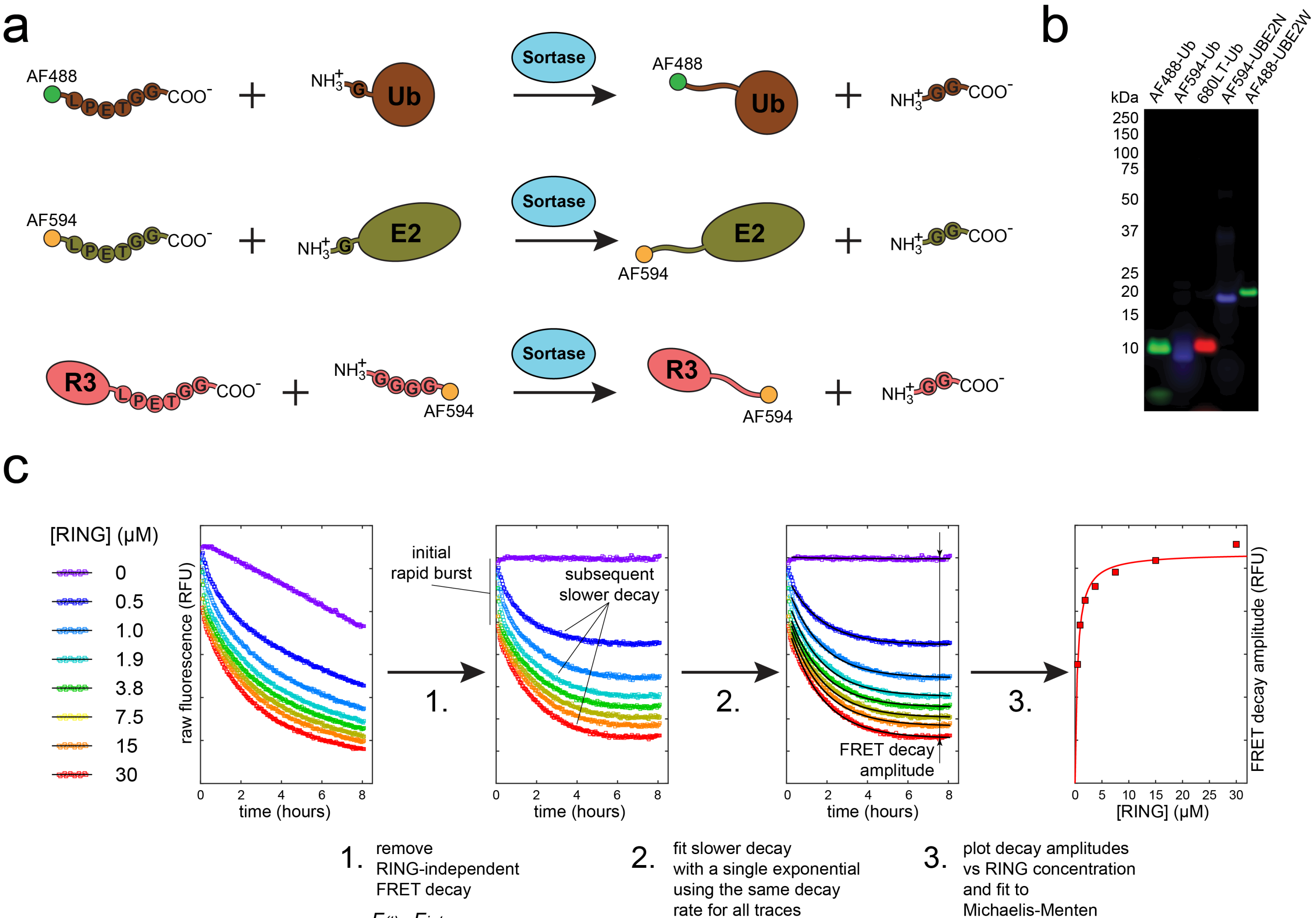

$$F'(t) = \frac{F(t) - F_{int}}{F^{RING=0}_{(t)} - F_{int}} + F_{int}$$

Supplementary Figure 2. Fluorescent labeling of proteins and analysis of UBE2W FRET decays. (A) Proteins were fluorescently labeled using sortase-catalyzed transpeptidation as described in Methods. (B) Fluorescent SDS-PAGE analysis of some fluorescently labeled proteins used in this study. (C) Analysis of FRET ubiquitin discharge data for the UBE2W~Ub conjugate. R3-WT dataset is shown as an example (Fig. 3C). First, the RING-independent component of FRET signal decay was removed from the datasets. FRET signals of the sample with no RING (purple trace) and the RING containing samples at later time points (6-8 hours) all appeared to decrease almost linearly towards a common intersection point (F_{int}). Therefore, this RING-independent contribution could be removed by applying one simple transformation to all datasets (step 1). RING-dependent component of FRET decay consisted of an initial rapid burst and a subsequent slower decay. The rate of the rapid burst was RING concentration dependent, but too fast to be determined reliably. Instead, the slower decay was fit to a simple exponential decay function (step 2). The rate of this slower decay was RING independent, whereas its amplitude increased with increasing RING concentrations. The dependence of FRET decay amplitude on RING concentration was fit to the Michaelis-Menten equation (step 3) and the initial slopes (the analogue of k_{cat}/K_m) were used to quantify the E3 activity of different RING constructs (Fig. 3D). Source data are provided as a Source Data File.

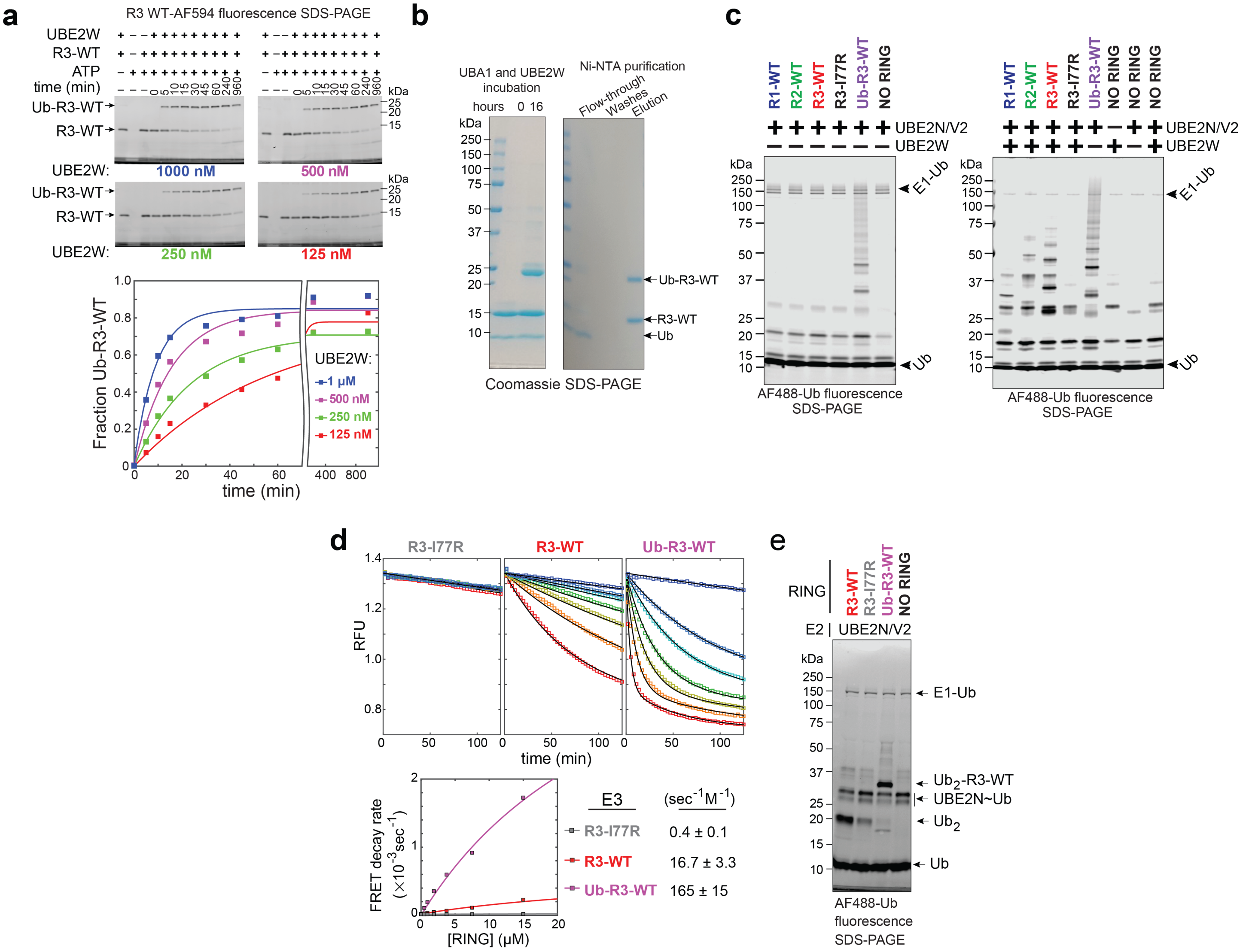

Supplementary Figure 3. Monoubiquitylation of R3-WT by UBE2W and its impact on the E3 activity of the RING trimer. (A) Fluorescent SDS-PAGE analysis of the R3-WT construct C-terminally fluorescently labeled with AF594 reveals that all three RINGs within the trimer are monoubiquitylated by UBE2W. [RING] = [R3-WT] = 1 µM; [Ub] = 10 µM; [UBE2W] as specified. (B) In vitro ubiquitylation of R3-WT ([R3-WT] = 50µM; [Ub] = 10 µM; [UBE2W] = 1 µM) and purification of the enzymatically ubiquitylated Ub-R3-WT product. (C) E3 activity of the enzymatically modified Ub-R3-WT and other RING constructs was evaluated by performing ubiquitylation reactions in the presence of fluorescently labeled ubiquitin and analyzed the products using direct fluorescent imaging of SDS-PAGE gel. [RING] = 1 µM; [Ub] = 5 µM; [UBE2W] = 0.1 µM incubated for 30 min. The Ub-R3-WT construct is significantly more active in its ability to catalyze polyubiquitin synthesis in the presence of UBE2N/V2, which indicates that monoubiquitylation activates UBE2N/V2-mediated polyubiquitin synthesis. (D,E) Measurement of the ubiquitin discharge kinetics by FRET (D) and analysis of the products by SDS-PAGE (E) revealed that the enzymatically modified Ub-R3-Ub displays a similar enhancement of E3 activity that was observed for the genetically modified Ub-R3-Ub (Fig. 4F-I). [UBE2N~Ub/V2] = 0.1 µM; [Ub] = 5 µM; [RING] as specified. Source data are provided as a Source Data File.

Primer name	Primer Sequence (5' to 3')
RING-monomer	CATAGTGGAGAAGCTCAGGGAGGTCAAGTTGAGCCCAGAATGAGCTT CTGGAATCCTGCT
RING-monomer I77R	CCTAATCGGCATGTAGCCAACCGCGTGGAGAAGCTCAGGGAGGTC
RING-monomer C-term FLAG	GAGCCCAGAAGAGGGAGATTATAAAGATGATGATGATAAATAACTCGA GCACCACCACCA
RING-dimer I77R 1st domain	AACATACAGCCTAATCGGCATGTAGCCAACCGCGTGGAGAAGCTCAG GGGATCCGCTTCT
RING-dimer I77R 2nd domain	AACATACAGCCTAATCGGCATGTAGCCAACCGCGTGGAGAAGCTCAG GGAGGTCAAGTTG
RING-dimer C-term FLAG	GAGCCCAGAAGAGGGAGATTATAAAGATGATGATGATAAATAACTCGA GCACCACCAC
RING-trimer I77R	AATATTCAACCGAACCGTCATGTTGCCAACCGCGTAGAAAAATTACGC GAAGTTAAATTA
RING-trimer, Tev site insert	CTACATTCCTGTCAGGCGAAAACCTGTATTTTCAGGGCCACCACCACC ATCATCATTAAG
RING-trimer, FLAG insert	CCTTTCTACATTCCTGTCAGGCGATTATAAAGATGATGATGATAAAGGC CACCACCACCATCATCATTAA

Supplementary Table 1. Primer sequences for RING constructs.

SUPPLEMENTARY METHODS

Data analysis of FRET ubiquitin discharge assays.

FRET-active E2~Ub conjugates described in this study enable studies of E3-catalyzed ubiquitin transfer kinetics. The assays described in Fig. 3 and Fig. 4F-I of the study are, in essence, a fluorescent implementation of ubiquitin discharge assays, in which the rate of ubiquitin discharge from the E2~Ub conjugates onto a substrate amine group is used to monitor the E3 activity. In contrast to traditional approach of visualizing ubiquitin discharge from E2~Ub by SDS-PAGE, we use the decay of the FRET signal to determine the ubiquitin discharge rate. Here the analysis of the FRET data is described in more detail.

In general, reactions catalyzed by E3 ubiquitin ligases are bisubstrate reactions, in which an E3 ligase (enzyme) catalyzes transfer of ubiquitin from a E2-Ub conjugate (substrate 1) onto a substrate protein (substrate 2).

Even though the traditional Michaelis-Menten model (1) of enzyme kinetics was developed for reactions with only one substrate,

$$E + S \underset{k_{-1}}{\overset{k_1}{\rightleftarrows}} ES \overset{k_{cat}}{\rightarrow} E + P \qquad (1)$$

the Michaelis-Menten analysis can be informative for reactions with two (or more) substrates because such reactions can often be reduced to the Michaelis-Menten model by keeping concentrations of additional substrates constant.

The Michaelis-Menten equation (2)

$$v = \frac{k_{cat}[E][S]}{K_m + [S]} \qquad (2)$$

describes the rate of the catalyzed reaction, where [E] and [S] denote total initial concentrations of the enzyme and the substrate in the reaction mixture. It is an approximation that is most accurate when [S]>>[E], which ensures that the concentration of free substrate is approximately equal to total substrate concentration. Traditionally, k_{cat} and K_m (or k_{cat} / K_m) values for a given enzyme-substrate combination are determined by measuring the reaction rate at different substrate concentrations and with [S]>>[E]. In most situations this is the most practical approach because the molecular weight of the substrate is much less than the molecular weight of the enzyme. However, this is not the case for ubiquitin discharge reactions described here where the RING constructs (enzyme) have lower molecular weight than E2~Ub conjugates (substrate 1). Furthermore, the E2~Ub conjugate is the most valuable reagent, and it is more practical to perform the reaction with the [E2~Ub] concentration kept constant and by varying [RING] concentration, and with [RING]>>[E2~Ub]. It is evident from the Michaelis-Menten model (1) that when the experiments are performed by varying E and having E>>S, the reaction rate is approximated by equation (3).

$$v = \frac{k_{cat}[S][E]}{K_m + [E]} \qquad (3)$$

Equation (3) can be used just like the Michaelis-Menten equation to analyze the dependence of the substrate depletion rate on enzyme concentration and to determine the k_{cat} and K_m values for an enzyme-substrate pair.

Another practical complication of using traditional Michaelis-Menten analysis for ubiquitin discharge kinetics is the problem of accurate measurement of the “initial rates” of a reaction. Using only the initial slope of a reaction progress curve for data analysis is suboptimal, because it relies on a small initial portion of the progress curve and discards the rest of the data thus reducing the overall accuracy of the measurement. [This data analysis strategy originated when computational resources were not readily available and data fitting was performed by drawing a straight line through the dataset or finding a tangent to a curve by eye.] In the traditional Michaelis-Menten analysis higher precision can be achieved when [S]>>[E] and [S]>>K_m, such that the depletion of the

substrate during the measurement does not significantly diminish the reaction rate (zero-order kinetics). However, E3s usually have relatively low affinity for E2~Ub conjugates (high K_m) and it is more practical to perform the reaction when both [E3] and [E2~Ub] are < K_m. In addition, it is also more practical to perform the experiments with [E]>>[S] as is explained above. It is evident from equation (3) that, when the substrate concentration is below K_m and [S]<<[E], substrate depletion is well approximated by an exponential decay function (4):

$$v = -\frac{d[S]}{dt} = \frac{k_{cat}[E]}{K_m + [E]}[S]$$

From which it follows that

$$[S](t) = [S]_0 * \exp\left(-\frac{k_{cat}[E]}{K_m+[E]} * t\right) \qquad (4)$$

It is a property of the exponential decay function y(x)=exp(-a*x) that the initial slope (first derivative at t=0) is equal to -a. Therefore, determining the value of -a by fitting the entire progress curve to exponential decay yields a more accurate value of the reaction velocity at t=0, than what can be obtained by trying to fit the initial slopes. Ubiquitin discharge from UBE2N/V2 is well approximated by an exponential decay function (Fig.3B), therefore we used this approach to analyze the UBE2N/V2 ubiquitin discharge data. It is evident from (4) that the dependence of the E2~Ub depletion rate on [RING] ([E]) concentration is described by the hyperbolic equation (analogous to the Michaelis-Menten equation), the initial slope of which is equal to k_{cat} / K_m. This is how the k_{cat} / K_m values reported in Fig. 3D were determined.

In contrast, ubiquitin discharge progress curves obtained with the UBE2W~Ub conjugates were not well described by the Michaelis-Menten model as is explained in the Results section (Fig. 3C). The UBE2W~Ub dataset illustrates limitations of the Michaelis-Menten analysis when applied to bisubstrate reactions. The initial rapid burst of FRET decay was followed by a slower decay phase, the amplitude of which, but not the rate, was dependent on the RING concentration. The mechanistic basis of this distinctive kinetics will require further study. Because the rate of the initial burst, albeit RING concentration dependent, was too fast to be measured accurately, the amplitude of the RING-dependent FRET decay was used instead (see Supplementary Fig. 2C). Dependence of decay amplitude on RING concentration displayed a good fit to the Michaelis-Menten equation, and the initial slopes were once again used as a quantitative measure of the E3 activity of different constructs (Fig. 3E).
