# Supplementary material for: Structural and functional asymmetry of RING trimerization controls priming and extension events in TRIM5α autoubiquitylation
Source: Nat Commun. 2022 Nov 19;13:7104. doi: 10.1038/s41467-022-34920-3 (PMC9675739; doi:10.1038/s41467-022-34920-3)
Supplement: Supplementary file 2 — Reporting Summary [file 41467_2022_34920_MOESM2_ESM.pdf]

nature research

Corresponding author(s): Dmitri Ivanov
Last updated by author(s): Oct 28, 2022

Reporting Summary

Nature Research wishes to improve the reproducibility of the work that we publish. This form provides structure for consistency and transparency in reporting. For further information on Nature Research policies, see Authors & Referees and the Editorial Policy Checklist .

Statistics

For all statistical analyses, confirm that the following items are present in the figure legend, table legend, main text, or Methods section.

n/a	Confirmed	
☐	☒	The exact sample size (*n*) for each experimental group/condition, given as a discrete number and unit of measurement
☐	☒	A statement on whether measurements were taken from distinct samples or whether the same sample was measured repeatedly
☒	☐	The statistical test(s) used AND whether they are one- or two-sided<br>*Only common tests should be described solely by name; describe more complex techniques in the Methods section.*
☒	☐	A description of all covariates tested
☒	☐	A description of any assumptions or corrections, such as tests of normality and adjustment for multiple comparisons
☒	☐	A full description of the statistical parameters including central tendency (e.g. means) or other basic estimates (e.g. regression coefficient) AND variation (e.g. standard deviation) or associated estimates of uncertainty (e.g. confidence intervals)
☒	☐	For null hypothesis testing, the test statistic (e.g. *F*, *t*, *r*) with confidence intervals, effect sizes, degrees of freedom and *P* value noted<br>*Give P values as exact values whenever suitable.*
☒	☐	For Bayesian analysis, information on the choice of priors and Markov chain Monte Carlo settings
☒	☐	For hierarchical and complex designs, identification of the appropriate level for tests and full reporting of outcomes
☒	☐	Estimates of effect sizes (e.g. Cohen's *d*, Pearson's *r*), indicating how they were calculated

Our web collection on statistics for biologists contains articles on many of the points above.

Software and code

Policy information about availability of computer code

Data collection	To collect the circular dichroism data, we used Spectra Manager V1.55.00.<br>To collect the NMR data, we used Bruker Topspin V3.2.<br>To collect the fluorescence data, we used BioTek Gen5 V3.10.<br>To collect the SDS-PAGE data, we used a TYPHOON TRIO Imager V5.0.<br>To collect the AUC data, we used XLI AUCProteaome Lab V1.0.
Data analysis	MATLAB R2019B (Mathworks) was used to analyze the fluorescence decay curves.<br>NMR data were processed using NMRPipe V8.7 and analyzed using NMRFAM-Sparky V1.413.<br>SEDFIT 15.2b and 16.1 was used to analyze the analytical ultracentrifugation data.<br>Western Blots were analyzed using ImageStudio Lite 5.2.

For manuscripts utilizing custom algorithms or software that are central to the research but not yet described in published literature, software must be made available to editors/reviewers. We strongly encourage code deposition in a community repository (e.g. GitHub). See the Nature Research guidelines for submitting code & software for further information.

Data

Policy information about availability of data

All manuscripts must include a data availability statement. This statement should provide the following information, where applicable:
- Accession codes, unique identifiers, or web links for publicly available datasets
- A list of figures that have associated raw data
- A description of any restrictions on data availability

Data supporting the findings of this paper are available from the corresponding authors upon reasonable request. Source data are provided with this paper. There is no structural data to deposit into a publicly available database. Full spectra of CD data was not acquired.

Field-specific reporting

Please select the one below that is the best fit for your research. If you are not sure, read the appropriate sections before making your selection.

☒ Life sciences ☐ Behavioural & social sciences ☐ Ecological, evolutionary & environmental sciences

For a reference copy of the document with all sections, see nature.com/documents/nr-reporting-summary-flat.pdf

Life sciences study design

All studies must disclose on these points even when the disclosure is negative.

Sample size	No sample size calculation was performed. The sample size was chosen based on previous experience with each specific assay. Most assays are highly reproducible when experiments are repeated on multiple occasions.
Data exclusions	No data was excluded from this study.
Replication	To verify the reproducibility of our data, we have done duplicates of our measurements. Multiple biophysical techniques under the same conditions produced the same findings. All attempts at replication were successful.
Randomization	No randomization was performed. Covariance are not relevant for our experiments.
Blinding	Blinding was not possible for experiments because sample preparation and sample measurements for a particular experiment were performed by the same individual.

Reporting for specific materials, systems and methods

We require information from authors about some types of materials, experimental systems and methods used in many studies. Here, indicate whether each material, system or method listed is relevant to your study. If you are not sure if a list item applies to your research, read the appropriate section before selecting a response.

Materials & experimental systems

n/a	Involved in the study	
☐	☒	Antibodies
☒	☐	Eukaryotic cell lines
☒	☐	Palaeontology
☒	☐	Animals and other organisms
☒	☐	Human research participants
☒	☐	Clinical data

Methods

n/a	Involved in the study	
☒	☐	ChIP-seq
☒	☐	Flow cytometry
☒	☐	MRI-based neuroimaging

Antibodies

Antibodies used	Anti-ubiquitin antibody; Supplier: Cell Signaling Technology; CATALOG #: 3933S.<br>Anti-K63 ubiquitin antibody; Supplier: Cell Signaling Technology; CATALOG #: 3496S.<br>Anti-FLAG antibody; Supplier: Millipore; CATALOG #: F1804.<br>Secondary Fluorescent antibody; Supplier: LICOR; CATALOG #: 926-3211 and 926-68073.
Validation	These are widely used and commercially available antibodies, described in over 1000 peer reviewed publications. More details regarding manufacturer's validation procedures can be found here: https://www.cellsignal.com/products/primary-antibodies/ubiquitin-antibody/3933, https://www.cellsignal.com/products/primary-antibodies/k63-linkage-specific-polyubiquitin-d7a11-rabbit-mab/5621, https://www.sigmaaldrich.com/US/en/product/sigma/f1804, https://www.licor.com/bio/reagents/irdye-800cw-goat-anti-rabbit-igg-secondary-antibody, and https://www.licor.com/bio/reagents/irdye-680rd-donkey-anti-rabbit-igg-secondary-antibody.
